# A generalized numerical model for clonal growth in scleractinian coral colonies

**DOI:** 10.1098/rspb.2024.1327

**Published:** 2024-09-13

**Authors:** Eva Llabrés, Eleonora Re, Naira Pluma, Tomàs Sintes, Carlos M. Duarte

**Affiliations:** ^1^ Institute for Cross-Disciplinary Physics and Complex Systems IFISC (CSIC-UIB), Universitat de les Illes Balears, Palma de Mallorca 07122, Spain; ^2^ Hawai‘i Institute of Marine Biology, University of Hawai‘i at Manoa, Kaneohe, HI 96744, USA; ^3^ Marine Science Program, Biological and Environmental Science and Engineering Division, King Abdullah University of Science and Technology (KAUST), Thuwal 23955-6900, Saudi Arabia

**Keywords:** clonal growth, scleractinian coral colonies, agent-based modelling, coral growth dynamics, generalized minimal model

## Abstract

Coral reefs, vital ecosystems supporting diverse marine life, are primarily shaped by the clonal expansion of coral colonies. Although the principles of coral clonal growth, involving polyp division for spatial extension, are well-understood, numerical modelling efforts are notably scarce in the literature. In this article, we present a parsimonious numerical model based on the cloning of polyps, using five key parameters to simulate a range of coral shapes. The model is agent-based, where each polyp represents an individual. The colony’s surface expansion is dictated by the growth mode parameter (*s*), guiding the preferred growth direction. Varying *s* facilitates the emulation of diverse coral shapes, including massive, branching, cauliflower, columnar and tabular colonies. Additionally, we introduce a novel approach for self-regulatory branching, inspired by the intricate mesh-like canal system and internode regularity observed in *Acropora* species. Through a comprehensive sensitivity analysis, we demonstrate the robustness of our model, paving the way for future applications that incorporate environmental factors, such as light and water flow. Coral colonies are known for their high plasticity, and understanding how individual polyps interact with each other and their surroundings to create the reef structure has been a longstanding question in the field. This model offers a powerful framework for studying these interactions, enabling a future implementation of environmental factors and the possibility of identifying the key mechanisms influencing coral colonies’ morphogenesis.

## 1. Introduction

Corals are colonial organisms composed of genetically identical polyps that collaborate to construct complex structures. Each polyp, an individual animal of the cnidarian family, contributes to the colony’s growth by asexually reproducing through division and secreting calcium carbonate to form the coral skeleton. This collaborative effort allows corals to form a wide array of shapes, ranging from compact, massive domes to intricate, highly branched structures [1]. The clonality of individual polyps is essential for colony growth and is the primary strategy driving the expansion of coral reefs, which support extensive marine biodiversity and sustain the livelihoods of an estimated billion people globally [2]. Although the basic principles of clonality are well established, our understanding of how polyps interact with each other and their environment to form such intricate and varied shapes remains limited [3–6]. This complexity arises from the interplay between genetic predispositions and environmental plasticity [7,8], highlighting a sophisticated balance that shapes the structures of coral colonies.

Despite the recognized importance of asexual reproduction in corals, the literature lacks comprehensive modelling efforts to study clonal coral growth, especially when compared to the extensive models developed for plants since the 1980s [9,10]. A limited number of coral growth forms and taxa have been described through mathematical modelling as reviewed in [6], mainly focusing on the environmental modulation of their shapes. The only series of studies in the literature addressing this topic aims to formulate an accretive growth model to examine the influence of water flow on Pocilloporidae corals [11–17]. However, a universal model of coral clonal growth, applicable across growth forms and scleractinian coral taxa, comparable to existing models of tree growth [18–21] is still lacking.

Numerical models serve as crucial tools for testing hypotheses and addressing specific questions regarding coral clonal developmental processes [22]. Similar to their applications in botany, the models for coral dynamics have the potential to enhance our understanding of growth patterns and the interactions among individuals, which give rise to emergent properties and complex colony structures [23]. They also help in understanding plasticity influenced by environmental interactions [7,24] density-dependent processes [25–27] and guiding restoration projects [28]. Given the critical role of clonal growth in coral reef recovery from disturbances [29] and the success of reef restoration initiatives [30], the development of generalized models for coral clonal growth could significantly contribute to guiding conservation and restoration efforts.

In this study, we develop a universal growth model for scleractinian coral clones that simulates the growth patterns of coral colonies. Our approach uses a parsimonious set of simple rules inspired by the cloning behaviour of the polyps, skeleton formation and self-regulating branching processes. We aim to simulate the deposition of calcium carbonate by polyps, which varies based on species and polyp location within the colony. The interaction among individual coral polyps to form complex and mesmerizing structures is an open area of research in the field. Influenced by both environmental factors and genetics, the high plasticity of coral colonies often makes it difficult to identify species and disentangle the relevance of these mechanisms in coral formation. Our objective is to create a framework capable of recreating coral shapes, which can be used in future studies to investigate the biological mechanisms underlying the various forms of coral colonies. Specifically, the present work focuses on the development and sensitivity analysis of the model, demonstrating that by varying only five parameters, we can replicate a wide range of coral colony shapes, from massive to branching forms. This model provides a foundational tool for further exploration into the environmental and genetic factors that drive the diversity in coral morphology.

## 2. Methods

**Figure 1.**
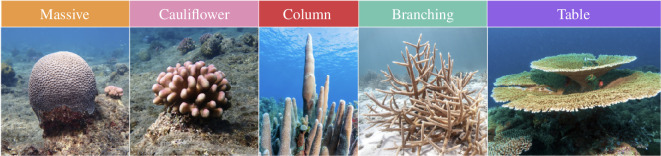
The pictures represent five of the most common and representative shapes of coral colonies found in reefs worldwide [1,31]. Credits for the images to Hugo Mann, johnandersonphoto and Rostislavv.

**Figure 2.**
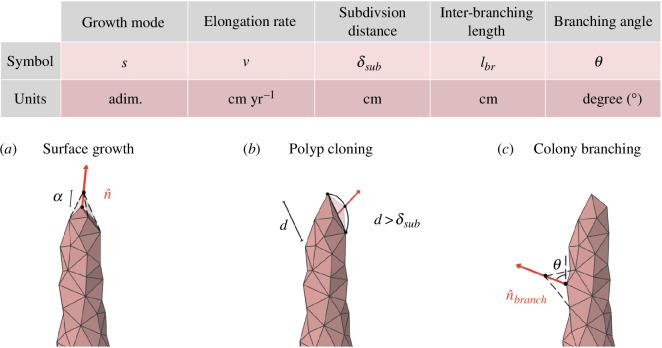
Table summarizing the relevant parameters of the coral colony model (top) and a graphic representation of its growth steps (bottom), where the vertices on the triangles represent the individual polyps. At each iteration, the following steps are repeated: (*a*) *Surface growth*: selected vertices will expand along their normal vector $\hat{n}$ , a distance α=ν Δt each time step Δt . The growth mode s determines which vertices are more likely to expand. (*b*) *Polyp cloning*: new clones are generated if the distances between vertices, d in the figure, are higher than $\delta_{sub}$ . The positions of the new vertices are located at the center of Bezier interpolation curves that unite the original vertices (represented by the black curved line), created using the Casteljau algorithm [32]. (*c*) *Colony branching*: polyps are selected and displaced a distance 4α over the vector ${\hat{n}}_{br}$ , which forms an angle θ with the primary branch. This perturbation generates new branches in the colony in the following iterations.

We developed an agent-based model to simulate scleractinian coral colonies, using individual polyps as agents. By employing a minimal set of geometric and biological parameters, the model accurately reproduces a wide range of existing coral colony shapes (figure 1). We employed a triangular mesh to depict the surface covered by the coral tissue, with each vertex corresponding to the location of an individual polyp (see figure 2). The structure filling the interior of the triangulated surface describes the calcium carbonate skeleton. To simulate the growth of the colony, we adjusted the positions of polyps based on three empirically derived processes: (*a*) surface growth, (*b*) polyp cloning, and (*c*) self-regulatory branching. Scleractinian coral colonies primarily grow owing to the continuous secretion of calcium carbonate by individual polyps, which expands the coral’s skeleton surface area (*a*). They also undergo a process of clonal growth known as asexual budding resulting in the formation of clones that occupy all the available interstitial spaces within the coral colony (*b*). In certain coral species, particular polyps have accelerated growth, both in terms of physical size and calcium carbonate deposition. This trait results in the regular branching structures distinctive of coral species such as *Acropora* (*c*). These mechanisms are implemented in our model at each time step Δt by following these clonal growth rules:

- The calcium carbonate accumulation by polyps is influenced by their position within the colony. This dependency is modelled through a selective displacement of mesh vertices, driven by the input model parameters $s_{min}$ and $s_{max}$ , which are dimensionless and range from 0 to 1. Each of the vertices is assigned a numerical value using the following auxiliary functional:
(2.1) $$\xi \left(\hat{n}\right)=\frac{2}{\pi }\arctan\left(1-\frac{n_{z}}{\sqrt{{\left(n_{x}\right)}^{2}+{\left(n_{y}\right)}^{2}}}\right),$$
  depending on their normal vector at the surface, defined as $\hat{n}=\left(n_{x},n_{y},n_{z}\right)$ , which is unitary. This function is designed such that its bounds either correspond to a complete vertical or horizontal direction, taking the values $\left(\xi =1;n_{x},n_{y}=0\right)$ and $\left(\xi =0;n_{z}=0\right)$ , respectively. The parameter s adjusts the preferred growth direction of the colony by applying the following: polyps will only expand if their associated ξ value falls between the interval $s=\left[s_{min},s_{max}\right]$ . Thus, the parameter s restricts the trajectories of polyps in the colony’s evolution with the constraint ξ<s . If this condition is met, a vertex at a three-dimensional point V will relocate to $V+\alpha \hat{n}$ during a time interval Δt (figure 2*a*). The parameter α represents a distance related to the linear elongation rate of the colony’s surface ν [cm yr^−1^] through α=ν Δt . Linear elongation rates vary based on coral shapes; for example, for branching corals, ν corresponds to the linear growth of the branches, whereas for massive corals, it represents the radial growth rate of the sphere. Additional details on linear elongation rates are provided in §3a.
- New clones are generated when the inter-polyp distances become large enough to accommodate new individuals. In this work, we define the inter-polyp distance as the distance between the centers of two neighboring polyps, which corresponds to the sides of the triangles in the mesh. At each iteration, we compare the sides of the triangles to a maximal inter-polyp distance $\delta_{sub}$ , in millimetres. If the distance among two vertices is greater than $\delta_{fuse}$ , a new polyp is added in between them using a surface interpolation as shown in figure 2*b*. Triangles connecting the new vertices are generated following a subdivision scheme similar to that proposed in [15]. In this step, vertices will also fuse if they are closer than a distance $\delta_{fuse}$ , which is defined as $\delta_{fuse}=0.2\;\delta_{sub}$ . The fusion procedure has the purpose of smoothing and eliminating unrealistic elongated triangles, but is also based on a natural process observed in coral colonies, where polyps must die or be reabsorbed [33,34].
- In certain coral colonies, selected polyps undertake specialized roles and initiate the formation of new side branches. This process is simulated in our model by selecting branch-leading vertices with a probability $p_{br}$ , and directing their expansion along a unitary vector ${\hat{n}}_{branch}$ . The vector ${\hat{n}}_{branch}$ is defined sharing the horizontal direction with $\hat{n}$ , while its vertical component forms an angle θ with the *z*-axis (figure 2*c*). To ensure a regular distribution of the branches, new leading polyps are selected from a subset of polyps at a distance $l_{br}\pm \epsilon$ from existing branching nodes, where $l_{br}$ represents the average measured inter-branching distances in natural colonies, and ϵ corresponds to its standard deviation. The probability $p_{br}$ for a polyp to start leading a new branch is not an independent parameter, since it is related to the inter-branching distance by $p_{br}=\nu \;\cdot {\left(l_{br}N_{br}\right)}^{-1}$ , where $N_{br}$ denotes the number of already existing branches. Furthermore, our implementation considers the branching of coral onto existing branches, where the new branch forms an angle θ with respect to the original branch.

Each step of our model is inspired by biological and mechanical processes occurring during coral morphogenesis. In step (a), we mimic the skeleton formation in coral colonies, where polyps secrete calcium carbonate at different rates depending on their species and their location within the colony’s surface. For instance, in pillar colonies, polyps preferentially secrete vertically, while in massive colonies, polyps secrete uniformly, creating a hemispherical shape [35]. In our model, the different growth modes are governed by the choice of the parameter s , which determines the preferred direction of expansion, and its variation allows us to reproduce various clonal shapes. In natural colonies, the emergence of specific shapes is influenced by a combination of genetic factors and environmental adaptations [36–38]. In this study, we focus on how the model can reproduce different shapes by varying s and creating a novel framework for, in the future, further studying the main mechanisms behind the specifics of each colony. While this is a purely theoretical manuscript, we refer the reader to §4a in the discussion for a potential experimental interpretation of the growth mode s .

Step (b) simulates the cloning process, inspired by polyp budding, which occurs through internal cues once polyps reach a certain size [39], and environmental responses when they have enough space available [40]. This is mimicked in our model by duplicating vertices when their distance exceeds $\delta_{sub}$ . In step (c), we model the branching process to resemble the ramification patterns appearing in the colonies of the *Acropora* genus. In these corals, branching occurs as a result of polyps taking specialized roles (i.e. polymorphism), making them larger and secreting more calcium carbonate that develops a lateral branch [41–44]. These colonies exhibit a certain regularity in the branching distance and angle [44], parametrized in our model by $l_{br}$ and *θ*, respectively. We have assumed that this process is a self-regulated branching mechanism, reminiscent of apical growth in plants. However, the exact biological processes regulating branching in corals have not yet been identified. For further discussion on the hypotheses and literature regarding the regulatory mechanisms of coral branching refer §4b.

The previous rules can be ultimately represented by a set of only five independent parameters (figure 2). The growth of colonies of various shapes in our study emerges as a consequence of iteratively applying these rules at each time step, commencing from an initial seed, a hexagonal pyramid (hexacone), which serves as a representation of the incipient calcium carbonate skeleton. Within the hexacone, the top vertex corresponds to the initial polyp of the colony, sexually produced, while the vertices at the base symbolize the first generation of clones. The choice of the hexaconal shape is drawn from the natural hexagonal organization observed in scleractinian corals, belonging to the hexacorallia class, where attributes such as the presence of tentacles, septa and mesenteries commonly occur in multiples of six. We chose the sides of the hexagonal base that have length $\delta_{sub}$ , and the height of the pyramid $\delta_{sub}/2$ , such the initial polyp and its neighbours start with reasonable separation distances. To fix the structures to the ground, movement of the vertices at the base is restricted to the horizontal plane, i.e. their growth is guided by a modified vector ${\hat{n}}_{base}$ , which represents the average normal of adjacent faces, as detailed in (a), with the vertical component suppressed.

In this article, we will not use empirical data to set the parameters in our simulations. Our main goal is to assess the validity of the model, and we will use approximations, values from previous literature or sweeping to study the parameter space. For example, in step (c), we defined the parameter ϵ *,* which represents the uncertainty within which a new branch can form while respecting an average branching distance $l_{br}$ . Ideally, ϵ should correspond to the standard deviation of $l_{br}$ on the measurements from actual colonies. However, for our purposes, we set ϵ to the reasonable value of $0.25\;l_{br}$ *,* which corresponds to a 25% measurement error. Additionally, the time step of the model has been fixed throughout this article at Δt=0.1yr . It is also necessary to mention that we made use of libigl, a C++ library designed for geometric processing. We employed some of its built-in functions, including those for computing average vertex normals and mean distances among neighbouring elements.

## 3. Results

The proposed numerical model, as introduced in §2 and visually depicted in figure 2, faithfully replicates the most characteristic coral structures using a remarkably concise set of only five biological parameters: the growth mode ( s ), the elongation rate ( ν ), the subdivision distance ( $\delta_{sub}$ ) the inter-branching length ( $l_{br}$ ) and the branching angle ( θ ). In this section, we present our findings, accompanied by a comprehensive exploration of the model’s sensitivity to variations in each of these parameters.

### (a) Reproducing real coral colonies: s and ν

**Figure 3.**
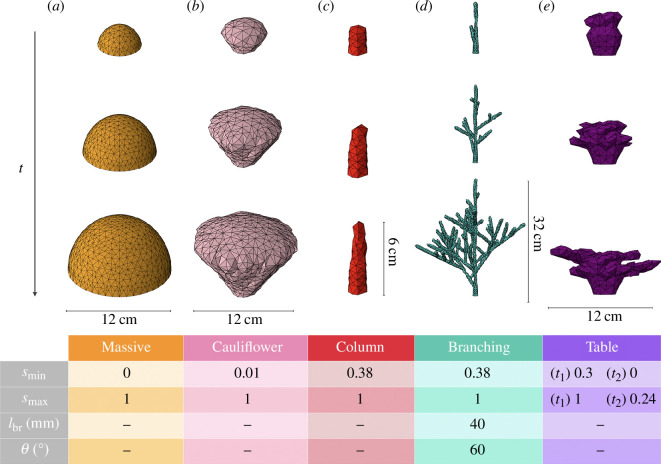
Time evolution of the structures generated with our model for different choices of the growth mode ( $s_{min}$ , $s_{max}$ ), the inter-branching length ( $l_{br}$ ) and the branching angle ( θ ). The drawings are organized into columns (*a*)-(*e*), each representing a specific combination of the parameters as detailed in the table at the bottom of the figure. The rows correspond to snapshots taken at three different times: t=12,22,32yr , for the branching and t=2,4,6 yr for the rest of the shapes. The other parameters of the model have been fixed to $\nu =10\;mm\;yr^{-1}$ and $\delta_{sub}=10\;mm$ in all cases. Exceptionally, the right-most structure is produced considering two growth regimes: an initial growth at $t_{1}=0\;yr$ guided by s = [0.30, 1] , which at time $t_{2}=3\;yr$ is modified to s = [0, 0.24] . This shift reproduces the characteristic transition from vertical to horizontal expansion that is observed in table corals. For a dynamic visualization of the evolution of the coral structures collected in this figure, see the electronic supplementary materials, videos S1–S5.

The parameters **$s_{min}$** and $s_{max}$ determine the principal direction of extension of the coral’s skeleton, playing the most crucial role in the model. These parameters are essential because they enable the reconstruction of a diverse range of shapes mirroring real-life coral configurations (figure 3). For example, the election s = [0, 1] guarantees uniform growth across all polyps, culminating in a semispherical form (figure 3*a*), representative of the massive corals observed in natural habitats (figure 1*a*). For a time-resolved visualization of the progressive evolution of the massive coral simulation, see the electronic supplementary material, video S1. Inhibiting the growth of predominantly horizontally oriented polyps with s = [0.01, 1] , yields a shape reminiscent of an inverted cone (figure 3*b*; electronic supplementary material, video S2), analogous to the cauliflower-shaped corals (figure 1*b*). The range s = [0.375, 1] represents a predominantly vertical expansion, effectively emulating the characteristic columnar coral morphologies (figures 1*c* and 3*c*; electronic supplementary material, video S3). In figure 3*d* and the electronic supplementary material, video S4, we replicate the branching coral configuration employing an identical s parameter, while also incorporating apical dominance by setting $l_{br}\;=\;4\;cm$ . Notably, this adjustment yields the intricate branching patterns characteristic of coral structures (figure 1*d*). Finally, we can also integrate two distinct growth modes, an initial columnar growth characterized by s = [0.375, 1] , subsequently transitioning towards a predominantly horizontal growth with s = [0.375, 1] . This combination of s is based on the growth dynamics of table corals as it is observed in nature [45] and allows us to replicate their morphology in figure 3*e* and the electronic supplementary material, video S5. While we can reproduce the evolving conical shape of the cauliflower corals, replicating their more complex branching structures observed in figure 1*b* remains a challenge. For additional comments on this issue, we refer to §4e.

The elongation parameter, denoted as ν , determines the quantity of calcium carbonate accumulated by each polyp, thereby exerting control over the temporal dynamics of colony growth in figure 3. In the context of massive and cauliflower-shaped corals, ν is intricately tied to the radial expansion of the sphere and cone, respectively, signifying the extent of the colony’s expansion radiating outward from the colony’s initial polyp. By contrast, the columnar and branching coral configurations are governed by an elongation ν that represents the linear extension rate of each of the branches. For the table colonies, the parameter ν times the radial extension of the top disc during the tabular regime. Different types of colonies have different experimental elongation rates: branching and tabular (Acroporidae family) $12\;mm\;yr^{-1}$ ; massive (Poritidae family) $7\;mm\;yr^{-1}$ ; and columnar (Agaricidae family) $8\;mm\;yr^{-1}$ [31,35]. However, we have chosen an equal elongation rate for all shapes in our simulations, $\nu =10\;mm\;yr^{-1}$ , which is within the expected range from observations. This choice has been made by simplicity reasons and adjusting ν to the exact experimental values will only modify the time steps required to reproduce a particular structure, but not its main shape.

Additionally, we would like to mention that we extended the evolution time of the branching shape to 32 yr, compared to 6 yr in the other simulations. This longer duration allowed us to achieve a taller structure (32 cm in height) which, given the higher complexity of the branching shape, was required for validating and performing the sensitivity analysis. Applying real elongation rates and developing different coral shapes for equal times would lead to branching colonies occupying more space than the rest of the species, as expected from field observations.

### (b) Polyp surface distribution: $\delta_{sub}$

**Figure 4.**
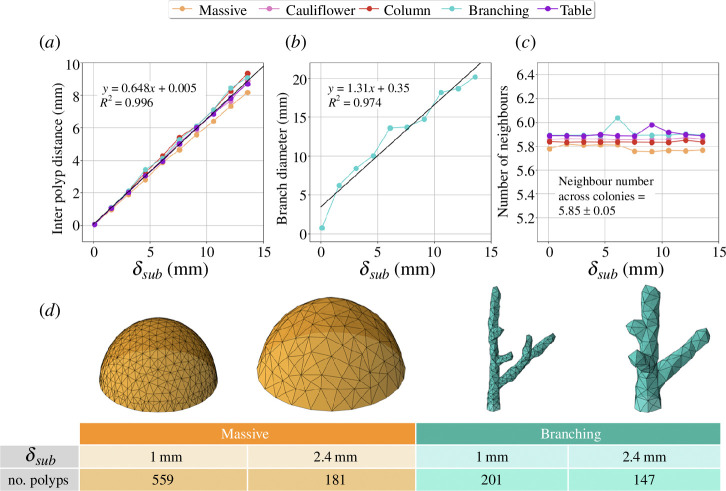
Sensitivity of the model to changes in the subdivision distance, $\delta_{sub}$ . (*a*) Linear relationship between the average inter-polyp distance and $\delta_{sub}$ for all colony shapes. (*b*) Branch diameters hold a linear relation with $\delta_{sub}$ . The diameters have been measured by averaging them at different positions and for different branches of branching colonies. (*c*) The average number of neighbours is close to six for all colony shapes, independent of the choice of $\delta_{sub}$ . (*d*) Snapshots exemplify different surface-filling distributions of polyps depending on $\delta_{sub}$ . All four depicted colonies in (*d*) have grown for a time of 10 yr. On the contrary, in graphics (*a*)–(*c*), the colonies have evolved until the measured value stabilizes. The elongation rate ν has been set to $10\;mm\;yr^{-1}$ , and the growth modes $s_{min}$ and $s_{max}$ are chosen following figure 3 to reproduce all colony shapes.

In figure 4, we explore the influence of the subdivision distance, $\delta_{sub}$ , on the various coral shapes. This parameter dictates the cloning process, triggering new clone formation when the distance between two polyps surpasses a certain threshold. It is logical to anticipate that $\delta_{sub}$ would influence the inter-polyp distance. Indeed, our analysis shows a stable linear relationship between $\delta_{sub}$ and the average inter-polyp distance that is valid across all colony morphologies, exhibiting a slope of 0.72 (figure 4*a*).

Furthermore, the applicability of our model spans several orders of magnitude of inter-polyp distances. As depicted in figure 4*a*, the model accurately replicates distances ranging from 0.07 to 9.5 mm, aligning closely with observed dimensions in colonies across various species [46–48]. In scenarios where the coral skeleton assumes a columnar or branching morphology, the variation of $\delta_{sub}$ is also related linearly to the change in the branch thickness, as shown in figure 4*b*. Remarkably, branch thickness is governed by the inter-polyp distance or $\delta_{sub}$ and remains unaltered irrespective of simulation duration. Each simulation depicted in figure 4*b* is carried out until reaching a steady state of branch thickness for every $\delta_{sub}$ .

The parameter $\delta_{sub}$ also determines the total number of polyps filling a given shape. Figure 4*d* shows how coral colonies that have grown the same amount of time, but at different $\delta_{sub}$ values, exhibit comparable morphologies while displaying distinct polyp counts populating their surfaces. This visual representation for massive and branching colonies exemplifies the applicability of the numerical model to represent different structures with various inter-polyp distances. It is also remarkable that vertices consistently exhibit an average of six neighbouring connections across all shapes and $\delta_{sub}$ , as depicted in figure 4*c*. These hexagonal patterns arise in our simulation from two key features of the model: the representation of shapes using a triangular mesh with polyps as vertices, and the application of a subdivision step based on polyp budding, where triangles divide when one of their edges is longer than $\delta_{sub}$ . This process results in a mesh of equilateral triangles that then organize into hexagonal lattice.

### (c) Exploring branching colonies: θ and $l_{br}$

**Figure 5.**
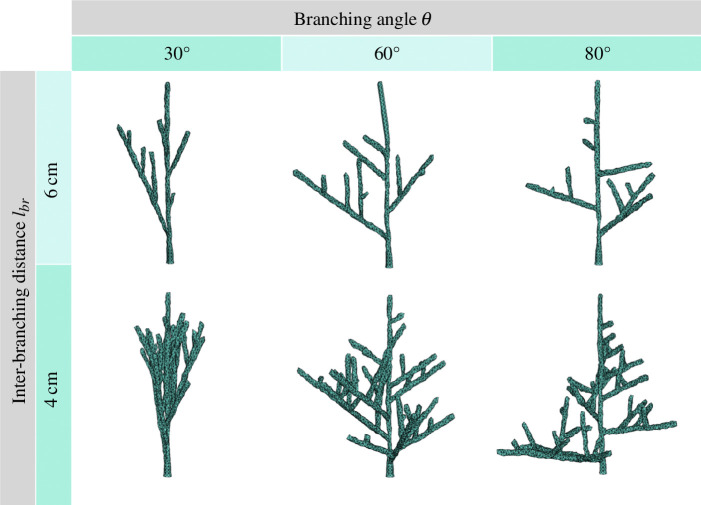
Branching coral structures reproduced with different choices of the model parameters. Columns correspond to a change in inter-branching distance $l_{br}$ and the column varies the branching angle θ . The rest of the parameters have been set to: $s\;=\;\left[0,\;0.375\right],\;\;\;\nu \;=\;10\;mm\;yr^{-1},\;\;\;\delta_{sub}=10\;mm$ and run for t= 32 yr . The three-dimensional objects representing these colonies are displayed in the electronic supplementary material, named as Object Sl _ θ , where l labels the inter-branching distance and θ the branching angles fixed for the specific shape.

Branching colonies are generated by connecting the parameters θ and $l_{br}$ to columnar shapes with s = [0, 0.375] . In figure 5, we present an array of choices for these parameters, shedding light on their impact on colony morphology. When $l_{br}$ is set to lower values, an increase in branching probability becomes apparent, resulting in the formation of denser coral colonies. The parameter θ governs the primary inclination of these branches, its values spanning the range from 0 to $90^{\circ }$ degrees. Smaller θ angles engender colonies that predominantly adopt a vertical orientation, as exemplified in the snapshot presented at the left of figure 5. Conversely, larger θ angles yield colonies that exhibit a more extensive spatial occupancy, as illustrated at the right of figure 5. These variations in $l_{br}$ and θ directly influence the spatial distribution of branches and contribute to the striking diversity in colony forms that can be achieved through the model.

### (d) Continuity of s and coral hybrids

As showcased in figure 3, our model describes a variety of coral shapes from a simple biologically inspired model that relies on a set of only five independent parameters. The resulting geometric diversity is mainly governed by the parameters $s_{min}$ and $s_{max}$ , which are adimensional, vary from 0 to 1, and dictate the primary direction of growth of the colony. The continuous range of allowed values for s facilitates a smooth transition across the spectrum of coral colonies. This progression is exemplified in the electronic supplementary material, figure S1, where we observe a transformation from massive to columnar shapes when increasing smoothly the value $s_{min}$ , and fixing $s_{max}=1$ . For the limit case $s_{min}=0$ , the initial seed of the simulation is allowed to grow both vertically and horizontally with equal probabilities, yielding a semispherical form (electronic supplementary material, figure S1a). The choice of values for $s_{min}$ greater than 0 marks a shift, anchoring the coral base owing to the exclusion of purely horizontal expansion. Consequently, this leads to the formation of shapes reminiscent of inverted cones, with the degree of opening narrowing as $s_{min}$ increases (electronic supplementary material, figure S1*b,c*). Eventually, complete vertical growth is attained, with only the topmost vertices undergoing expansion (electronic supplementary material, figure S1*d,e*). Conversely, a distinct transformation occurs when $s_{min}$ is held constant at 0 while $s_{max}$ is varied, as illustrated in the electronic supplementary material, figure S2. As $s_{max}$ diminishes below zero, the semispherical shape begins to exhibit flattening at the top (electronic supplementary material, figure S2*b,c*). This progressive flattening continues until $s_{max}$ reaches 0, where growth becomes exclusively horizontal, resulting in the reproduction of encrusting coral forms (electronic supplementary material, figure S2d).

In the electronic supplementary material, figures S1 and S2, we systematically explored the complete space of values for $s_{min}$ and $s_{max}$ that lead to the development of a mature coral structure. Any other combination of these parameters, in which $s_{min}\neq \;0$ or $s_{max}\neq \;1$ , fails to generate a coral colony structure, remaining in the initial state of the hexagonal seed. This outcome is primarily attributed to the initial seed structure, characterized by the top polyp exhibiting solely vertical growth and the base polyps being constrained to horizontal growth exclusively. If $s_{min}\neq \;0$ or $s_{max}\neq \;1$ , the initial vertices are unable to expand in any of the iterations. However, it is possible to generate other coral shapes by transitioning from one growth mode to another during the growth process, following a methodology inspired by our approach to table corals in figure 3. This development is exemplified in the electronic supplementary material, figure S3, where various combinations of growth modes are showcased. Furthermore, we introduced branching possibilities by connecting the parameters θ and $l_{br}$ to growth modes that deviate from the columnar structure, as illustrated in the electronic supplementary material, figure S4. The results presented in the electronic supplementary material, both figures S3 and S4 correspond to geometries that are not typically found in natural coral colonies, and we refer to them as *coral hybrids*.

## 4. Discussion

We developed a numerical model that replicates the growth patterns of the main coral colony shapes observed in nature, using only a set of five basic parameters (figure 3). Massive coral colonies emerge from our simulations when all growth directions are equally likely, cauliflower coral colonies appear as vertical growth starts to exceed horizontal growth, and columnar coral colonies appear when vertical growth is predominant, in contrast with tabular growth, which appears when there is a shift from an initial vertical growth to a later horizontal expansion [45]. Finally, branching corals arise from columnar colonies when apical dominance enhances branching at a given distance of already existing branches. Also, the model preserves its features and allows for coral colony representations for different polyp sizes and inter-polyp distances (figure 4). That the broad diversity in realized coral colony shapes can be effectively captured with such a limited number of parameters is remarkable, and it will serve as the starting point for the development of advanced models that incorporate environmental factors as it is done for universal models of tree growth [18–21].

### (a) Polyp trajectories and the growth mode (*s*)

Our model is built to reproduce the accretion of calcium carbonate by coral polyps, represented by each of the vertices of a triangular mesh. This approach effectively connects the micro-spatial scale of individual polyps with the resulting macroscopic shapes exhibited by coral colonies. Accretive models were proposed in earlier works [11,15,17,48], from which we inspired our subdivision schemes for polyp asexual budding (see §2, step (b)). These studies focused on reproducing colonies with a cauliflower morphology, including hydrodynamic effects to replicate the geometries of species such as *Pocillopora verrucosa*. However, these earlier works did not account for a key factor to the universality of our model: the parameter *s* that modulates the growth of the colonies to achieve a wide variety of shapes, which range from predominantly vertical to horizontal. The inclusion of this parameter represents a significant advancement towards a universal model of coral clonal growth, addressing a notable gap in existing literature and reproducing several coral colony shapes from a single numerical model.

The parameter s is described theoretically in this article, but there are methods to validate its applicability using experimental data. In this paragraph, we outline a potential approach. In step (a) in §2, we defined the magnitude ξ , which assigns a value to each polyp that relates to its expansion direction. For ξ tending to 1, the polyp expands in a direction close to vertical, and for ξ tending to 0, the main direction is horizontal. The parameter s adjusts the preferred growth direction of the colony by applying the constraint that polyps will only expand if ξ<s . Thus, s restricts the trajectories of polyps in the colony’s evolution. To determine if s has an interpretation in real-life colonies, we propose using computed tomography (CT) scans of coral skeletons [49,50]. CT scans reveal canal-like structures in coral skeletons that trace the history of polyp trajectories within the colony. We hypothesize that by assigning ξ to each of these trajectories, based on their normal directions and using equation (2.1), we can reproduce the constraint ξ<s across the different coral growth forms analysed in this article (figure 3). This hypothesis is currently being tested in ongoing research.

In this article, the parameter s is chosen to be positive, reflecting the vertical growth tendency of most corals, which orient themselves with the seabed as their reference point. This upward growth pattern is crucial for several reasons. Primarily, corals grow vertically to optimize light exposure, an essential factor for their symbiotic relationship with photosynthetic algae, which require adequate sunlight to perform photosynthesis effectively [38]. Furthermore, this growth strategy can be linked to gravitaxis mechanisms, similar to those observed in plants, where organisms grow directionally in response to gravitational forces [51]. This directional growth ensures that corals can extend towards nutrient-rich water layers and away from potential sediment accumulation that can smother and inhibit their growth. Additionally, vertical growth may help corals avoid competition for space and resources on the seabed, facilitating the formation of complex, three-dimensional reef structures that support diverse marine ecosystems. Despite these insights, the precise biological and environmental cues driving this vertical growth remain not fully understood and more comprehensive studies in this regard are needed.

### (b) Apical dominance and the importance of colony integration

In this article, we introduce a novel modelling technique for a self-regulated branching process in coral colonies (see §2, step (c)), wherein a selected polyp undergoes faster growth, leading the direction of a new branch. This mechanism is inspired by the biological development observed in finely branched species, exemplified by the Acroporidae family, as documented in [41–43]. Our approach parallels the concept of apical branching in plants, where apical buds, typically located at the tips of shoots, dominate the growth and direction of branches. This modelling technique diverges from the method used in [16], whose simulations produced coral morphologies resembling *Pocillopora* and *Stylophora* species. These species exhibit rounder and less sharp branches without a dominant apical polyp guiding branch formation. Indeed, *Stylophora* has been shown not to possess apical dominance [52], while empirical evidence to confirm apical dominance in *Acropora* species is currently lacking. Understanding whether *Acropora* species exhibit apical dominance could provide significant insights into their growth patterns and adaptive strategies, offering broader implications for coral reef ecology and conservation.

A particular characteristic of the *Acropora* genus is the presence of a highly intricate mesh-like canal system connecting all polyps within the colony [53]. By contrast, species like *Pocillopora* exhibit isolated polyps connected solely through their living tissue or coenosarc [54,55]. The connectivity observed among polyps in *Acropora* species is hypothesized to represent a higher evolutionary feature, contributing to the emergence of complex and expansive coral structures when compared to the bounded branching forms of *Pocillopora* [24]. The association between intricate three-dimensional structures and functional connectivity among polyps has been proposed in earlier studies [56,57], but there exists a gap in the literature regarding its relationship to apical branching. The inner canals function akin to gastrovascular systems, elevating individual polyps to a higher organizational level, analogous to the relationship between cells in plant or tree structures. Consequently, we hypothesize branching in *Acropora*-like species is possibly driven by chemical signals flowing through the canal system connecting the polyp. This reasoning is also inspired by shoot branching processes in flowering plants, regulated by hormonal factors transported through their vascular system [58–60].

### (c) Hexagonal patterns in scleractinian corals

Distinctive features of a real coral colony appear organically through the clonal rules inherent in our model, without the need for predetermined inputs. As illustrated in figure 5*c*, the simulated polyps exhibit an average number of 5.8 neighbours, faithfully replicating the natural hexagonal symmetry characteristic of hexacorals [47,60]. Our model consistently maintains and reproduces this hexagonal symmetry irrespective of inter-polyp distance or colony shape, controlled by parameters $\delta_{sub}$ and s , respectively. The hexagonal lattice in our simulation arises from two key features of the model: the representation of shapes using a triangular mesh with polyps as vertices, and the application of a subdivision step based on polyp budding. This process results in a mesh of equilateral triangles that organize into a hexagonal lattice. In nature, this behaviour probably derives from polyps’ adaptive strategy to maximize coverage on a given surface, as the hexagonal lattice is the densest circle-packing configuration of the plane [61]. Our simulations show a neighbour count slightly below six, a departure probably attributed to the non-planar nature of coral skeletons. To account for the positive curvature on surfaces and preserve the densest circle packing, pentagons must be included in the tiling, as exemplified in the geometry of a truncated icosahedron [62].

### (d) Environmental factors and plasticity

Establishing a comprehensive model that incorporates all coral shapes is crucial for discerning the primary mechanisms governing coral growth. Moving forward, our research will expand to encompass the modulating effect of environmental factors, such as light and nutrient availability, on the parameters determining clonal growth. The parameter *s*, currently employed as a mechanistic variable for creating morphologies, could be extended in future research to encompass a combination of environmental and genetic factors influencing colony growth. For instance, by introducing a light source into our simulations, we could vary the calcium carbonate accumulation among polyps based on their position within the colony and the amount of light they receive. This approach would enable us to replicate field observations of coral colonies that alter their morphology in response to light [63,64]. Additionally, water flow and nutrient diffusion could be implemented in the simulation to affect colony growth, similar to the approach in [11–17]. The role of polyp interactions in shaping coral morphologies has been a longstanding inquiry in the field [45,65]. Our model offers a powerful framework to address this question, identifying the key mechanisms influencing coral colonies and distinguishing between genetic and environmental factors.

The inclusion of diverse environmental factors in our model is pivotal for evaluating the susceptibility of specific coral species to changing conditions, forecasting future reef scenarios and assessing the efficacy of replantation campaigns. Drawing inspiration from the work in Cresswell *et al*. [66], who proposed a growth model featuring non-plastic coral colonies represented through schematic pixel art, we aim to investigate how different regions of the reef population respond to variations in environmental variables. By incorporating environmental factors into our model, we can conduct similar studies while also considering the plasticity of individual colonies and its impact on the overall coral reef ecosystem. This comprehensive approach promises to enhance our understanding of coral dynamics and contribute to more effective conservation and management strategies.

Our research aligns closely with the field of *theoretical morphology,* which aims to understand why certain biological forms exist while others do not [67]. This discipline uses mathematical models with minimal parameters to generate both hypothetical and real forms, significantly contributing to our understanding of evolution. In our study, parameters such as the growth mode ( s ) and branching length and angle ( $l_{br}$ and θ ) act as axes of a morphospace, allowing us to simulate both real coral forms (figure 3) and hypothetical coral hybrids (electronic supplementary material, figures S3 and S4). Applying our model in the context of theoretical morphology could provide valuable insights into the mechanisms behind coral shape formation and explain why certain forms are favoured in natural environments over others. The unique shapes observed in coral hybrids, which are not typically found in natural coral colonies, suggest that these forms may confer reduced fitness and are thus selected against. Factors contributing to this reduced fitness could include shading that impairs polyp growth, diminished hydrodynamic flow and nutrient delivery or instability in high-energy environments. Therefore, examining how different coral colony shapes interact with their environment could elucidate why these particular hybrid shapes are absent in nature.

### (e) Limits to the generality of the model and future improvements

The parsimonious universal model of coral clonal growth developed here offers a novel tool for investigating space occupation by corals, their interactions with the environment, and even for advancing the design of restored coral reefs and generating unique three-dimensional printed colonies—a technology gaining traction for coral restoration [50,68]. However, several improvements are necessary before these applications can be fully realized. One evident limitation of the current model is its inability to reproduce the open-branched forms typical of certain coral species. While we successfully matched the evolving conical shape in cauliflower coral, replicating the more complex branching forms observed in figure 1*b* remains a challenge. Specifically, the branching process, outlined in §2, step (*c*), did not yield the desired structure when we imposed the characteristic conical shape of the cauliflower, as observed in the coral hybrids (see the electronic supplementary material, figure S4b). The branching mechanism in cauliflower coral differs significantly from that in *Acropora* species. In *Acropora*, dominant polyps extend laterally to form new branches, resulting in horizontally expansive structures. By contrast, cauliflower corals exhibit more rounded branches without a predominant leading polyp, leading to more compact growth patterns. This difference in branching strategies necessitates additional features in the model to more accurately reproduce cauliflower coral shapes. We propose that incorporating an accretive growth process, similar to the one used in the works [11–17], could help address this limitation. Such a mechanism could be integrated into our model’s framework, given the similarities in the triangulated mesh extension and subdivision schemes employed in both studies. This integration could enhance the model’s capability to simulate the diverse morphologies of coral species more accurately, thereby improving its use for both ecological studies and practical applications in coral reef restoration.

To more accurately replicate coral colony structures, it is essential to introduce stochasticity into the model parameters. This addition would result in coral colonies that are less symmetrical and exhibit more irregular, realistic shapes. Additionally, for the branching corals reproduced in figure 5, we have not yet implemented mechanisms that allow different branches to collide in space more realistically. Such interactions occur in real Scleractinian corals. For example, some species exhibit intra-colonial branch fusion, known as anastomosis, which is particularly common in table *Acropora* [69]. Conversely, other coral species, like *Stylophora pistillata*, develop a buffer zone around each branch, preventing branches from encroaching on each other’s space [70]. In our current simulations, branches do not collide owing to the limited number of branches that appear in our simulations (we refer to Objects Sl _ θ in the electronic supplementary material). However, incorporating these processes is necessary to study more densely packed colonies and understand the characteristic horizontal extension observed in *Acropora*-dominated coral reefs. This would provide a more comprehensive understanding of coral colony dynamics and improve the realism of our models.

## Data Availability

Data and code are available on Dryad [71]. Supplementary material is available online [72].
